# Salmagundi

**Published:** 1885-01

**Authors:** 


### SALMAGUNDI.

EDITED BY E. G. BETTY, D.D.S.

HYDROCHLORATE of cocaine is worth *only* $180 an ounce.

AN electrical tooth-pulling machine, which is quick and almost painless in its operations, can pull as many as seven teeth in five seconds.—*Clipping.*

A PITTSBURG, PA., paper says that a Philadelphia man swallowed his false teeth and finds his digestion greatly improved. It seems to us that we have heard that story before.

MURIATE OF COCAINE.—NASHVILLE, December 13.—The students in the Dental Department of Vanderbilt University to-day witnessed the first application of the new local anæsthetic, muriate of cocaine, ever applied to dentistry. Several patients were operated on with success.—*Cin'ti Com. Gazette.*

Kempton.—Robert Paul, only son of Dr. Wm. D. and Jennie B. Kempton, died at 5 A. M., Saturday, December 13, 1884, aged 3 years and 12 days. Thus read the sad announcement in the newspaper. Dr. Kempton, our friend and classmate at both medical and dental colleges, has our sincerest sympathy in his bereavement; but, death awaits us all and our time comes sooner or later.

---

A correspondent, writing to one of our Eastern newspapers, says: "I am not a dentist, but I think people should become aware of the fact—that is, young and middle-aged persons who are losing their sight and hearing—that frequently the decay of these faculties is caused by bad teeth. When the teeth are pulled sight and hearing are restored."

Though a little far-fetched, may be, there is still a goodly proportion of truth in the above.

---

A good deal of the success in making a plate of Watt's metal depends upon the mixture with which the piece is invested. Dr. Sillito recommends two parts white sand to one of plaster, as that gives a more solid mass and one not so liable to crack when the flask is heated up, previous to pouring. Dr. Will Taft uses pulverized pumice instead of sand, mixing in about equal parts. This, he says, is much superior to sand, is smoother and will stand considerable heat without warping or cracking, a point of much practical importance.

---

Dr. B. Bettman, of Chicago, thus tabulates the physiological action and therapeutical effects of muriate of cocaine:

1. It is a powerful local anæsthetic, not penetrating in nature, rapid in its effects, which, however, are only temporary.

2. It is a mydriatic, the effect of which is regulated by the strength of the solution.

3. It produces paralysis of the ciliary muscle, the near point receding from the eye. Distant vision is not influenced.

4. By virtue of its benumbing powers it may be classified as an anodyne.

So far there has been nothing but benefit to those who use the morning porridge as a palatable and healthful change from the cakes made of buckwheat and other grains, which were formerly consumed in far greater quantities than since oatmeal made its appearance to share the favor then, and still, accorded them. Kind nature has dealt so lavishly with us, in the variety and excellence of the cereal products, that we have not to depend upon any single one of them to round out the pleasures of the morning meal to their fullest proportions. But oatmeal, nevertheless, has given us a new and wholesome dish for which we are duly appreciative.—*N. Y. Commercial Advertiser.*

---

As civilization advances, remarks the *Hotel Gazette*, the man who uses the quill toothpick gradually retires. This man, when he has finished the atrocious work of gouging his teeth, keeps the quill in his mouth and talks through it. He makes some sage like remark and then sucks the quill as though he would draw back his words. If he should be standing and you should be occupying a similiar position, he will approach, poke his face under the brim of your hat and whistle his words in your face. Then he will take out the quill and flip the corn bread off the point, put it back into his mouth and whistle a remark shrill and commonplace. Then he will chew the quill and make it snap and pop—take it out again, flip off a piece of beef muscle and—throw it away? No, sir; he puts it in his mouth and crunches it. He may be a man of high standing; he may be the monolith of a church, or the solid column of a temple of justice, but with all, he is a heathen.

---

Editor "Salmagundi:"

Have you ever seen a cuspid with two roots? Yesterday I prepared the mouth of Mrs. T. for plates by removing all the remaining teeth and parts of teeth. Among them were the two inferior cuspids, each of which have two well-defined roots, the separation being complete fully one-third the entire length of the tooth. I deem them curiosities, and will present one of them to the Ohio College of Dental Surgery, through the Dean. Any one wishing to see the anomaly, will, therefore have an opportunity. The position of the roots is labio-lingual, and they are nearly of the same length and size. If any one has found an irregularity of this sort, we shall be glad to hear from him.

COVINGTON, KY., Dec. 10, 1884. J. M. CLYDE, D.D.S.

Yes, we have seen just such a case, but only one, though it was one better in this that the *four* cuspids all had double roots. A medical friend asked us, several years ago, to replace the teeth in a skull he was preparing for a museum. Upon examination it was found that the inferior cuspids were divided nearly half the length of the tooth, and each root had its foramen at the apex. The upper ones were divided but an eighth of an inch beyond the apex, and with but one pulp canal, the depression at the bifurcation gradually disappearing towards the neck. Dr. Clyde's specimen is a very good one, and he did well to place it in the College Museum.

---

The following clipping from a New Orleans letter shows how in one way quacks bring disrepute upon our profession, though the writer (a layman) is intelligent enough to perceive the humbuggery:

"Fakirs" and quacks do a thriving business here on Sunday morning. How Dickens would have reveled in French market! On one side of the market last Sunday were two humbugs of the first water. They were both doctors. One was a colored "Hoodoo" and the other was a dispenser of a cure-for-all-pain. As an advertisement, the latter extracted teeth by a lightning process, free of charge. In about ten minutes I saw half a dozen men, women and children mount his stand to have troublesome masticators jerked out. The skill of the fellow was really wonderful. Laying the victim back in a chair, and resting the head in his arms, he rubbed his cure-all around the gum with his fingers, then he whisked a devilish looking instrument into the mouth—a tug or a twist, and in the twinkling of an eye the tooth was out, and dripping with blood, it was held up in triumph to the gaze of the spectators. From the commencement of the operation to the end an infernal machine in the rear kept up a racket by beating a drum and grinding from an organ the cheerful air of "Mollie

Darling." One man had four teeth pulled in succession. When the professor yanked out the first he looked at it admiringly for a second, and then said "one," and threw it among the crowd; another jerk, and the Professor said "two," and pitched it in the throng; and the third and fourth were treated in the same way.

